# Baseline circulating IL-17 predicts toxicity while TGF-β1 and IL-10 are prognostic of relapse in ipilimumab neoadjuvant therapy of melanoma

**DOI:** 10.1186/s40425-015-0081-1

**Published:** 2015-09-15

**Authors:** Ahmad A. Tarhini, Haris Zahoor, Yan Lin, Usha Malhotra, Cindy Sander, Lisa H. Butterfield, John M. Kirkwood

**Affiliations:** University of Pittsburgh Cancer Institute, UPMC Cancer Pavilion, 5150 Centre Avenue (555), Pittsburgh, PA 15232 USA

**Keywords:** Melanoma, Ipilimumab, CTLA4, Neoadjuvant, Cytokines

## Abstract

**Background:**

We evaluated candidate circulating serum cytokines, chemokines and growth factors in patients with locally/regionally advanced melanoma receiving neoadjuvant ipilimumab with toxicity and clinical outcome.

**Methods:**

Patients were treated with ipilimumab (10 mg/kg IV every 3 weeks, 2 doses) before and after surgery. xMAP multiplex serum testing for 36 functionally selected cytokines and chemokines was performed at baseline and at six weeks (following ipilimumab). Based on our prior data, the association of IL-17 and immune related colitis was tested. Serum cytokines were divided into functional groups (Th1, Th2, Regulatory, Proinflammatory) and were assessed at baseline and week 6 using sparse-group Lasso modeling to assess the association of various cytokine groups with progression free survival (PFS). The linear combination of the cytokines/chemokines in this model was then used as a risk score and a Kaplan-Meier curve was generated to examine the association of the dichotomized score and PFS.

**Results:**

Thirty-five patients were enrolled whose staging was: IIIB (3; N2b), IIIC (30; N2c, N3), IV (2). Median follow-up was 18 months. Among 33 evaluable patients, median PFS was 11 months (95 % CI = 6.2–19.2). IL-17 was found to correlate significantly with the incidence of grade 3 diarrhea/colitis when measured at baseline (*p* = 0.02) with a trend towards significance at 6 weeks (*p* = 0.06). In the modeling analysis, at baseline, the linear combination of 2 regulatory cytokines [TGF- β1 (*ρ* = 0.19) and IL-10 (*ρ* = -0.34)] was significantly associated with PFS (HR 2.66; *p* = 0.035). No significant correlations with clinical outcomes were found in examining the week 6 cytokines.

**Conclusions:**

Baseline IL-17 level was significantly associated with the later development of severe diarrhea/colitis while the combination of baseline TGF- β1 and IL-10 levels were associated with therapeutic clinical outcome after neoadjuvant ipilimumab. These findings warrant further investigation and validation.

**Trial registration:**

ClinicalTrials.gov Identifier NCT00972933.

## Background

Patients with clinically detectable lymphatic metastasis (American Joint Committee on Cancer stage IIIB-C) carry a poor prognosis with a risk of relapse and death that approaches 70 % at 5 years [1–3]. Complete surgical excision and regional lymph node dissection followed by adjuvant therapy with high dose interferon alfa-2b (HDI) has been the standard of care for this group of patients [1]. The host immune response is known to be relevant to disease outcome in melanoma and host cytokine and cellular immune responses may potentially be used to predict prognosis and response to therapy [4–6]. A better understanding of the regulation of the host effector and suppressor cellular and cytokine elements in response to melanoma may provide valuable mechanistic clues that may be exploited to predict therapeutic response to immunotherapy, including treatment with ipilimumab.

Ipilimumab is a human immunoglobulin-G (Ig G1k) anti- cytotoxic T-lymphocyte-associated protein (CTLA)-4 antibody which has been approved by the United States Food and Drug Administration (FDA) for the treatment of advanced inoperable melanoma based on phase III trial results [7]. However, treatment is associated with significant immune related adverse events and durable therapeutic benefits appear to be confined to a subgroup of patients. Therefore, it is of critical importance to define biomarkers that may predict clinical response and the risk of toxicity in patients treated with ipilimumab [8]. Assessment of these biomarkers in peripheral blood is particularly desirable given the facile accessibility, and ability to perform highly standardized repetitive assessments of blood biomarkers.

We conducted a neoadjuvant evaluation of ipilimumab administered at 10 mg/kg for patients with locally/regionally advanced melanoma, where we have previously reported significant clinical activity and immunomodulatory changes in the circulation and the tumor microenvironment with ipilimumab [9]. In this report, we evaluated a group of functionally selected cytokines and chemokines divided into 6 subgroups, and their association with clinical outcome. IL-17 is one of the central inflammatory cytokines upregulated in inflammatory bowel disease [10]. Therefore, we tested the association of IL-17 with the risk of immune mediated colitis after neoadjuvant ipilimumab.

## Patients, materials and methods

### Patients

All eligible patients were 18 years or older with clinically detectable local and/or regional melanoma (of cutaneous, mucosal or unknown primary site of origin). The Institutional Review Board of the University of Pittsburgh approved the study and the written informed consent that was obtained from all patients participating in the study.

### Study design and treatment

Patients were required to receive two doses of ipilimumab at 10 mg/kg intravenously given 3 weeks apart (induction). Surgery was planned 6–8 weeks after the initiation of ipilimumab,neoadjuvant therapy. After recovery from surgery, 2 additional doses of ipilimumab 3 weeks apart were planned (maintenance). Blood specimens for correlative studies were planned at baseline and 6 weeks (before surgery)—and then at 3, 6, 9 and 12 months or at progression.

### Toxicity and response assessments

For Adverse Event (AE) reporting, the description and grading scales of NCI Common Terminology Criteria for Adverse Events version 3.0 were used. Imaging studies were carried out at baseline, after 6–8 weeks of therapy (before surgery) and then at 3 months intervals for response assessment. Responses were not confirmed due to performance of definitive surgery to render the patient free of disease.

### Statistical methods

The original study design and statistical plan have previously been published [9]. This report compares the correlative cytokine and chemokine blood levels studied among patients who experienced diarrhea/colitis and those who did not, using the Wilcoxon sum rank test. Given the exploratory nature of this study, no adjustment for multiple testing was done—and the nominal *p*-values are reported. Sparse group Lasso (SGL) modeling analysis was used to evaluate the association of different subgroups of the 36 functionally selected cytokines (assessed at baseline and at week 6) with progression free survival (PFS). Cytokine levels were scaled so that all cytokines have the same scale. This method allowed us to simultaneously fit the model and select the markers and group(s) associated with benefit as defined by PFS. More importantly, it allows the incorporation of prior functional group information into the modeling. The functional group information was defined by the biological function as shown below. A regular Cox proportional hazard (CoxPH) model was fitted to the markers selected by SGL. The linear combination of the cytokines (i.e. the product of the cytokine level and the CoxPH model coefficients) was then used as the risk score. We dichotomized the risk score at the median and generated Kaplan-Meier (KM) survival curves to examine the association of the risk score and PFS.

Baseline descriptive statistics were performed on all evaluable patients for demographic variables, laboratory parameters, toxicity and therapeutic efficacy in relation to disease PFS. PFS were estimated by the Kaplan-Meier method.

### Laboratory methods and corresponding statistical analyses

#### Blood processing

Red top vacutainer tubes (no anticoagulant) were used for serum collection and all samples were processed within 24 h of collection (samples received before 5 pm were processed upon receipt, those arriving after 5 pm were processed the following morning). Serum samples were centrifuged at 2500 rpm for 10 min at 4 °C according to laboratory standard operating procedures (SOPs) and single use aliquots of each patient’s sera were then stored at -80 °C. The laboratory freezers were monitored continuously for any temperature fluctuations, and maintained the samples at -80C.

#### Multiplex serum cytokine analysis

Functionally selected 36 serum cytokines were tested. These included Th1 type cytokines (IFN-γ, IL-12 (p40/p70), IL-15, IL-17, IL-2, IL-7, IP-10), Th2 (IL-13, IL-5, IL-4), proinflammatory (IL-1α, IL-1β, IL-6, TNF-α, IL-1RA, IL-2R, IL-8, CRP, IL-17, IFN-α), immunoregulatory (TGF-β1, IL-10, PGE2), growth factor (VEGF, G-CSF, EGF, HGF, FGF-basic, GM-CSF), and other/chemokines (CCL5/RANTES, CCL3/MIP-1α, CCL4/MIP-1β, CCL2/MCP-1, CXCL9/MIG, CCL11/Eotaxin). The xMAP serum assay for these cytokines was performed according to the manufacturer’s protocol (BioSource International (Camarillo, CA)) as previously described [11] and laboratory SOPs, and analyzed on the Bio-Plex suspension array system (Bio-Rad Laboratories, Hercules, CA). Experimental data was analyzed using five-parametric curve fitting and assay controls included kit standards and multiplex QC controls (R & D Systems). Inter assay variabilities for individual cytokines were 1.0 to 9.8 % and intra-assay variabilities were 3.6 to 12.6 % (information provided by Biosource International and validation performed in our laboratory).

## Results

### Patient characteristics, treatment details, efficacy and safety

Thirty five patients were enrolled between 2/2010 and 10/2012. Eight patients had newly diagnosed melanoma whereas 27 patients had recurrent disease after treatment that included surgery. Twenty nine patients had cutaneous primary tumors, five mucosal and one with unknown primary melanoma. Eighteen patients had *in-transit* metastatic melanoma. On retrospective review, two patients demonstrated stage IV disease at baseline determined through the progression of previously questionable/undetected findings [9]. By stage, patients were classified as IIIB (3; N2b), IIIC (30; N2c, N3) and IV (2). Patient demographics and baseline disease characteristics were previously published [9]. A median of 4 cycles of ipilimumab per patient were administered with a total of 106 cycles [9]. Efficacy data has been previously reported [9]. Briefly, the median follow-up for patients at risk of progression was 17.6 months and for patients who were still alive was 16.1 months. The median PFS was 10.8 months, 95 %-CI (6.2, 19.2). The probability of PFS at 6 and 12 months was 0.72, 95%CI (0.53, 0.84) and 0.47, 95%CI (0.29, 0.63). The probability of survival at both 6 and 12 months was 0.97, 95%CI (0.78, 0.99). AEs related to ipilimumab have been reported previously [9]. These include the immune related AEs that were considered related to ipilimumab. Twenty patients (57 %) experienced diarrhea/colitis, including 9 (26 %) grade 1, 6 (17 %) grade 2 and 5 (14 %) grade 3. There were no grade 4 or higher events [9].

### Association of IL-17 level and diarrhea/colitis

**Fig. 1:**
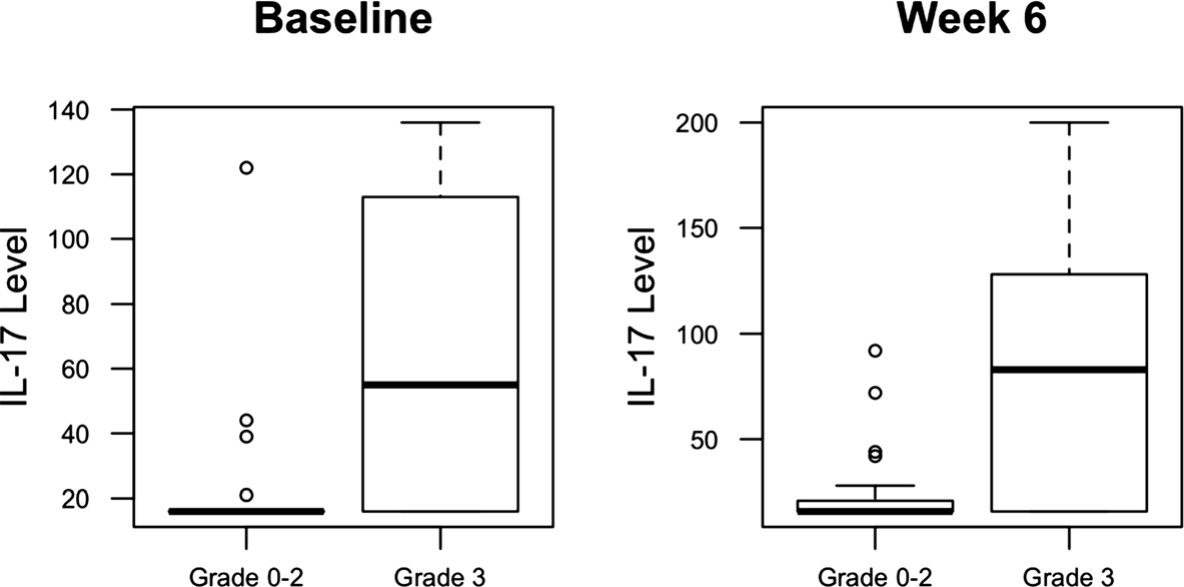
Serum levels of interleukin-17 (IL-17) in patients treated with neoadjuvant ipilimumab correlate Gr3 diarrhea/colitis when tested at baseline (*p* = 0.02) and at week 6 (*p* = 0.06)

To identify potential circulating biomarkers of efficacy and/or toxicity, serum samples were tested for levels of a broad array of analytes. Blood IL-17 levels at baseline were found to correlate significantly with the incidence of grade 3 diarrhea/colitis (*p* = 0.02), while this association remained with a trend towards significance at 6 weeks (*p* = 0.06). Figure 1 IL-17 was not significantly associated with all grades of colitis. We found an association between baseline IL-17 and any grade 3 irAE (similar to colitis, which was not surprising given that over 50 % of patients with irAE events had colitis), but the association was slightly less significant (*P* = 0.03). When we examined all grade irAE (including colitis, rash, hepatitis, endocrinopathies, pancreatitis), there was no significant association.

### Association of cytokines at baseline with PFS

**Fig. 2:**
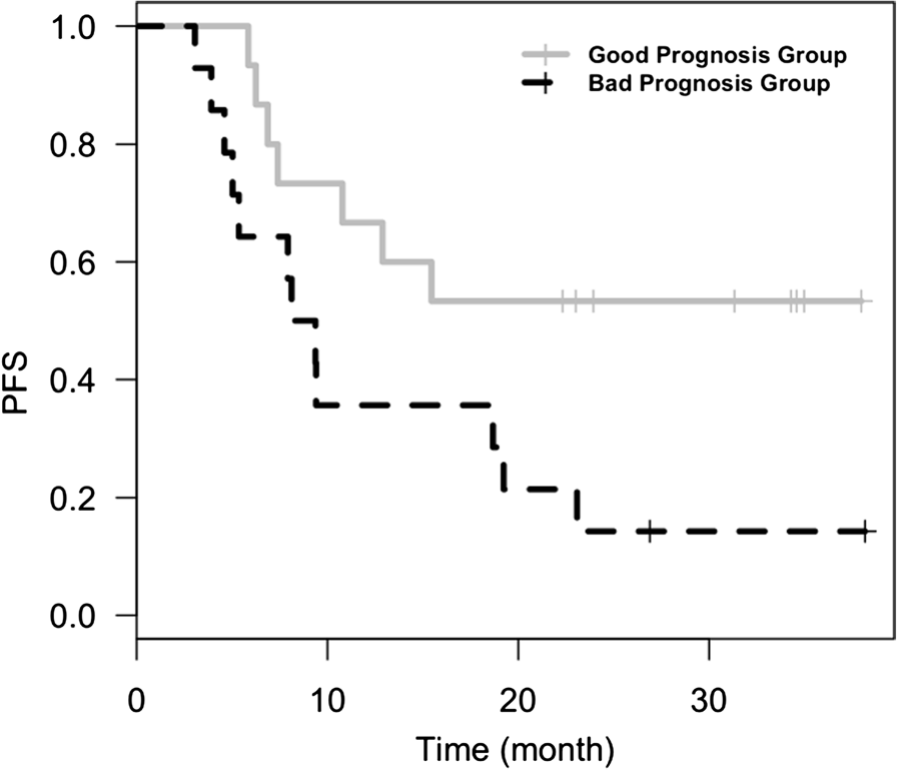
Baseline serum levels of TGF-β1 and IL-10 correlate with progression free survival (PFS)

The examination of individual cytokines with clinical outcome did not reveal any significant associations with disease outcome. Previous studies have identified groups of analytes as signatures of clinical outcome measures [12–16]. Therefore, we grouped our data into general functional groups based on their characterized roles in immune modulation as listed in Materials and Methods. In modelling analysis only one group of cytokines (the regulatory group) was selected by SGL as significantly associated with PFS. With further examination of these data grouped functionally, we identified a model with TGF-β1 and IL-10 as providing the best fit. IL-10 appeared to be a risk marker for progression, while unexpectedly, TGF-β1 appeared to be a marker of non-progression. The coefficients for the association of these two markers with PFS were 0.18 and -0.32 respectively. The dichotomized risk score based on these 2 markers was significantly associated with PFS (*p* = 0.036, HR = 2.66) as shown in Fig. 2. None of the type I cytokines tested correlated with clinical outcome.

### Association of cytokines with PFS at 6 weeks

The examination of the correlation of blood levels of individual cytokines with clinical outcome did not reveal significant associations. Similarly, the sparsed group lasso analysis results did not reveal any combination of markers that significantly correlate with PFS.

## Discussion

Ipilimumab has been shown to improve the survival of patients with advanced melanoma, but benefits only a subset of patients. Predicting the likelihood of clinical benefit from ipilimumab or the risk of developing toxicity remains an elusive goal. There is a critical need to define a biomarker or a set of biomarkers that can predict clinical response and the risk of toxicity in patients receiving ipillimumab. Major efforts in biomarker studies are ongoing and preliminary data are very encouraging, including gene expression signatures [9, 17], exome sequencing studies [18], and CD8 expression within the tumor microenvironment [19]. In this study, we evaluated the baseline and on-treatment serum levels of cytokines that have been functionally selected. The association of these blood cytokines with toxicity and clinical benefit after ipilimumab is novel and of potential clinical importance for this first systemic therapy to be approved in metastatic melanoma on the basis of survival benefits. Such an analysis is an important component of evaluating the host immune response to melanoma and may provide valuable mechanistic data, as well as allowing the improved prediction of benefit for patients who are considered for treatment with ipilimumab.

IL-17 is one of the central inflammatory cytokines and has been shown to be upregulated in the blood of patients with inflammatory bowel disease [10]. Therefore, the evaluation of IL-17 in patients with ipilimumab-associated inflammatory colitis is well justified. Pairwise comparisons of serum IL-17 levels in patients with colitis (*n* = 13) compared to those without immune related AEs (irAEs) (*n* = 16) demonstrated significantly higher serum IL-17 levels in patients with colitis at week 7 (*p* = 0.007) and week 12 (*p* = 0.02) [20]. In the present study, we observed a significant correlation between the risk of subsequent development of grade 3 immune-mediated diarrhea, for the first time with circulating blood IL-17 assessed at baseline, among patients entering neoadjuvant therapy for regionally advanced melanoma. We also confirmed the prior reported association of elevated levels of IL-17 on treatment at week 6, with the development of grade 3 immune-mediated diarrhea (grade 3 was the worst grade obesreved in our study). The occurrence of irAEs has been reported to be associated with the overall clinical benefit in patients receiving ipilimumab [21–26]. This supports the notion that these AEs are related to the mechanism of action of ipilimumab, and its reversal of immune tolerance. The ability to predict a patient’s risk for developing irAEs such as autoimmune colitis may significantly impact clinical care. These data support further evaluation of the blood levels of this cytokine as a predictor of risk for this AE. Such predictive testing for inflammatory colitis may be most productive in combination with other serologic and genetic markers of inflammation known to be associated with inflammatory bowel disease in the population. To our knowledge, no associations between other cytokines and toxicity have been reported in the past.

Melanoma patients have been shown to have a different pattern of serum cytokines when compared to healthy controls [12, 27]. Yurkovetsky et al, reported a higher level of IL-1α, IL-1β, IL-6, IL-8, IL-12p40,IL-13, GM-CSF, MCP-1, MIP-1α, MIP-1β, IFN-α, TNF-α, EGF, VEGF, and TNF-RII in the plasma of melanoma patients compared with healthy controls [12]. High serum levels of IL-1β, IL-1α, IL-6, TNF-α, and chemokines MIP-1α and MIP-1β measured before treatment (HDI) were positively associated with RFS [12]. High levels of VEGF and fibronectin have been associated with lack of clinical response to high dose IL-2 therapy and worse overall prognosis in metastatic melanoma and renal cell carcinoma patients [28].

TGFβ is a well-known cytokine associated with immunosuppression [29] Blockade of TGFβ has shown therapeutic potential in preclinical tumor models and in clinical trials [29]. Our observation that higher serum TGFβ is associated with lack of tumor progression is quite unexpected. In contrast, we find that high baseline IL-10 levels to be correlated with tumor relapse. While TGFβ can have immune suppressive effects *in vitro* and in certain model systems *in vivo* [29], this observation may represent the presence of an ongoing protective immune response which is being endogenously counter-regulated in a TGFβ dependent fashion. This may be similar to our observations that increased Treg in the circulation is a positive prognostic marker (while Treg in tumors is a negative prognostic biomarker) [9]. However, our observation is not unique. A recent study reported that chemotherapy-responsive metastatic melanoma patients had higher serum TGFβ1 levels compared with chemotherapy-unresponsive patients (*p* = 0.05) [30]. In addition, patients with elevated serum TGF-β1 concentrations had a trend towards a favorable overall survival outcome compared to those with lower levels (median 30.1 vs. 20.9 months, respectively) in that study [30]. Similarly, high serum TGF-β1 level was found to be associated with improved survival in patients with breast cancer [31]. Therefore, this finding warrants further investigation.

We grouped serum cytokines functionally and conducted a modeling analysis in order to evaluate the linear combination of combinations of multiple cytokines belonging into four different functional groups, and to evaluate the impact of ipilimumab more rationally. The findings of our study should be taken as hypothesis-generating since further studies are needed to validate our findings and to refine our understanding of the role that readily assessed circulating peripheral blood cytokines may play in predicting the clinical impact of immunotherapy in patients with melanoma, both toxic and therapeutic. As with any assessment of serum or plasma-based biomarkers, pre-analytic variables of time between blood draw and processing, tube type, as well as storage conditions and freeze/thaw cycles can impact results. Our single institution study utilized a CAP/CLIA central laboratory which participates in external proficiency testing and which has substantial expertise in this area to reduce as many variables as possible. The ongoing intergroup phase III trial E1609 led by ECOG-ACRIN in which ipilimumab is being evaluated as postoperative adjuvant therapy for melanoma at 3 mg/kg and 10 mg/kg in comparison to high dose interferon-α provides an opportunity to further investigate and validate these results.

## Conclusions

In patients with regionally advanced melanoma who enrolled in a trial of neoadjuvant therapy with ipilimumab at 10 mg/kg, baseline pretreatment IL-17 is here for the first time shown to be significantly associated with the risk of subsequent development of severe immune-mediated diarrhea. The baseline levels of TGFβ1 and IL-10, as a dichotomized risk score based on these 2 markers, are significantly associated with PFS. The observed prognostic role of high TGFβ1 levels (unlike those of IL10) is supported by other reports in the literature. These findings warrant further investigation and confirmation in larger trials.

## Abbreviations

CRP: C-reactive Protein
CCL: Chemokine (C-C motif) ligand
ECOG: Eastern Cooperative Oncology Group
ACRIN: the American College of Radiology Imaging Network
EGF: Epidermal Growth Factor
FDA: Food and Drug Administration
HDI: High dose interferon alfa-2b
IL: Interleukin
IFN: Inferferon
GM-CSF: Granulocyte Macrophage Colony-Stimulating Factor
MCP-1: Monocyte Chemoattractant Protein-1
MIP: Macrophage Inflammatory Protein
PFS: Progression Free Survival
TGF: Transforming Growth Factor
TNF: Tumor necrosis factor
VEGF: Vascular Endothelial Growth Factor
